# 5-Methyltetrahydrofolate Is a Crucial Factor in Determining the Bioaccessibility of Folate in Bread

**DOI:** 10.1021/acs.jafc.2c03861

**Published:** 2022-10-07

**Authors:** Fengyuan Liu, Minnamari Edelmann, Vieno Piironen, Susanna Kariluoto

**Affiliations:** Department of Food and Nutrition, University of Helsinki, Agnes Sjöbergin katu 2, FI-00014 Helsinki, Finland

**Keywords:** faba bean, wheat, bread, baking, yeast, 5-methyltetrahydrofolate, folate bioaccessibility, folate stability, oxidation

## Abstract

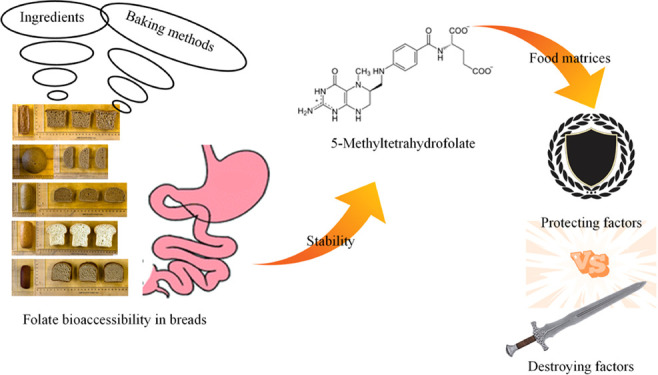

This study investigated the bioaccessibility of folate in wheat bread baked with different ingredients and processing methods. Next, different matrices were spiked with 5-methyltetrahydrofolate, gallic acid (GA), or both to investigate the stability of 5-methyltetrahydrofolate during in vitro digestion. The folate bioaccessibility in bread varied from 44 to 96%. The inclusion of whole-grain or faba bean flour significantly improved both folate content and bioaccessibility. Baking with yeast increased the folate content by 145% in bread but decreased folate bioaccessibility compared to the bread without added yeast because of the instability of 5-methyltetrahydrofolate. Spiking experiments confirmed oxidation as a critical reason for 5-methyltetrahydrofolate loss during digestion. However, GA protected this vitamer from degradation. Additionally, 5-methyltetrahydrofolate was less stable in whole-grain wheat matrices than other matrices. This study demonstrated that the stability of 5-methyltetrahydrofolate is crucial for folate bioaccessibility in bread, and methods for stabilizing this vitamer should be further studied.

## Introduction

Folate is an umbrella term describing a series of compounds that have similar structures and biological activities as folic acid. Folate is essential for the metabolism of amino acids, as well as for DNA and RNA synthesis.^1,2^ However, dietary folate intake in European populations is often insufficient.^3^ Deficiency of folate is associated with a series of developmental, immune, and neurological disorders, such as neural tube defects and anemia.^4^ Because humans cannot synthesize folate, mandatory fortification of bread flour with folic acid is practiced in several countries^5^ and shown to be efficient for preventing neural tube defects.^6^ However, mandatory folic acid fortification might cause excessive folic acid intake among population, especially if dietary folic acid supplements are taken at the same time.^7^ Excessive folic acid intake may mask a deficiency of vitamin B_12_.^8^ In addition, excessive ingestion of folic acid can cause a high level of unmetabolized folic acid in serum, which has been associated with the development of certain types of cancer, such as colorectal cancer.^9,10^ Therefore, it may be safer for the general population to consume sufficient folate from natural foods rather than from fortified foods.

Unlike folic acid, natural folates are usually chemically labile^11−13^ and can easily break down during digestion. Indeed, folate vitamers can undergo degradation and interconversion with oxygen and pH changes during digestion.^14^ Thus, the concepts of bioaccessibility and bioavailability were introduced to explain the proportion of folate ready for human absorption and utilization, respectively, in foods.^15^ In vivo experiments are needed for bioavailability studies, while in vitro digestion models are usually used for bioaccessibility research. Folate bioavailability varies considerably in different food matrices, ranging from 10 to 98%.^16^ In addition, a recent study investigated the bioavailability of folate in four different types of foods (custard, pudding, sponge cake, and biscuits) made with the same ingredients. The results demonstrated that the food structure can significantly affect folate bioavailability in humans.^17^

Because bioavailability studies are expensive and time-consuming, bioaccessibility studies are useful for generating hypotheses and screening samples. However, studies on the bioaccessibility of food folate are still rare. Öhrvik et al.^18^ reported that around 80% of folate in bread was bioaccessible. Mo et al.^19^ reported 82% folate bioaccessibility for tofu and approximately 100% for tempeh. Bationo et al.^20^ found that folate bioaccessibility varied from 23 to 81% in seven cereal-based fermented foods from West Africa. Ringling and Rychlik^14^ investigated the folate bioaccessibility of three food matrices (cheese, spinach, and wheat germ), and wheat germ had the lowest bioaccessibility at around 30%. Additionally, by studying the stability of individual folate vitamers during digestion, their results supported the idea that both the food structure and folate stability affect folate bioaccessibility.

The variation in the folate bioaccessibility data indicates that the stability of folate vitamers during digestion varies in different food matrices. Bread is an important source of dietary folate that is widely consumed around the world. Our previous research reported common trends in folate stability during in vitro digestion, and the degree of folate changes depended on the bread matrices.^21^ Because mainly commercial bread types were analyzed in our previous study, details of the processing were largely unknown, and thus, the effect of ingredients and processing methods on folate bioaccessibility in bread could not be confirmed. In addition, 5-methyltetrahydrofolate—one of the main folate vitamers in many foods^22^—was shown to be unstable in most bread matrices that we studied, and its content was negatively associated with folate bioaccessibility.^21^ We also showed that 5-methyltetrahydrofolate was more stable in faba bean matrices than in cereal matrices.^23^ Therefore, the aim of this study was to: (1) investigate the effect of ingredients and baking methods on the bioaccessibility of folate and (2) examine the varied stability of 5-methyltetrahydrofolate in different matrices during digestion.

## Materials and Methods

### Enzymes and Calibrants

α-Amylase from *Aspergillus oryzae* (A9857), pepsin (P7125), chymotrypsin (C4129), trypsin (T0303), protease (P8811), bovine and ovine bile (B8381), and gallic acid (G7384) were obtained from Sigma-Aldrich (St Louis, MO). (6*S*)-Tetrahydrofolate (H_4_folate, sodium salt), (6*S*)-5-methyltetrahydrofolate (5-CH_3_-H_4_folate, calcium salt), (6*R*,*S*)-5,10-methenyltetrahydrofolate hydrochloride (5,10-CH^+^-H_4_folate), and (6*S*)-5-formyltetrahydrofolate (5-HCO-H_4_folate, sodium salt) were purchased from Merck Eprova AG (Schaffhausen, Switzerland). 10-Formylfolic acid (10-HCO-PGA) and folic acid (pteroylglutamic acid, PGA) were purchased from Schircks Laboratories (Jona, Switzerland). 10-Formyldihydrofolate (10-HCO-H_2_folate) was prepared from 5,10-CH^+^-H_4_folate according to our previous protocol.^24^ The concentrations of the calibrants were checked spectrophotometrically. The preparation of the working calibrant solution was the same as in our previous study.^21^

### Baking Procedure

Whole-grain wheat flour (ash content 1.5–2.0%; Myllyn Paras, Hyvinkä, Finland), wheat flour (all-purpose wheat flour, ash content 0.6–0.7%; Pirkka, Järvenpä, Finland), and faba bean flour (Vihreä Härkä, Kalanti, Finland) were purchased from local markets. The ingredients for baking were flour, fresh yeast (Suomen Hiiva, Lahti, Finland), salt, sugar, liquid rapeseed oil (Bunge Finland Oy, Raisio, Finland), and water. Five types of bread were baked: whole-grain wheat bread, steamed whole-grain wheat bread, whole-grain wheat bread with no added yeast, white wheat bread, and faba bean wheat bread. The recipes used are provided in Table 1.

**Table 1:** Recipes for the Bread in the Baking Experiment

ingredient (g)	whole-grain wheat bread (WB)	steamed whole-grain wheat bread (SB)	whole-grain wheat bread with no added yeast (WNB)	shite wheat bread (WWB)	faba bean wheat bread (FWB)
wheat flour				500	250
whole-grain wheat flour	500	500	500		
faba bean flour					250
water	350	350	350	300	200
salt	7.5	7.5	7.5	7.5	7.5
sugar	10	10	10	10	10
yeast	25	25		25	25
fat	30	30	30	30	30
baking powder			10		

**Figure 1:**
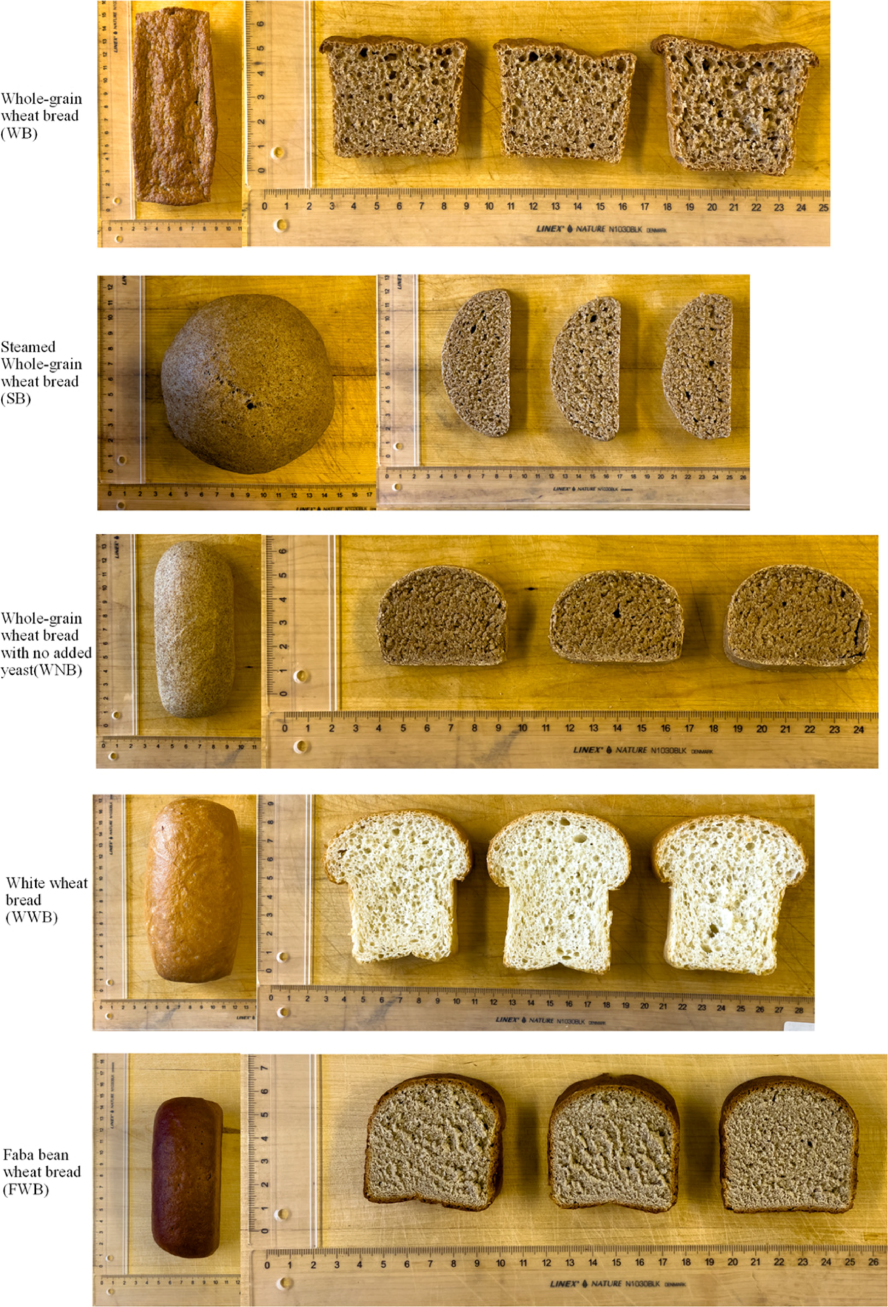
Bread types baked in this study.

The baking started by mixing the ingredients in a DIOSNA mixer bowl (Dierks & Söhne GmbH, Niedersachsen, Germany) for 3 min at low speed and then for 4 min at fast speed. Next, the dough was preproofed in a fermentation cabinet (Lillnord, Odder, Denmark) for 15 min at 35 °C and a relative humidity (RH) of 75%. After the preproofing, the dough was divided into three pieces (150 g each) and molded by a molder (EURO2000, Bertrand Puma, Nevers, France). The loaves were then proofed in molds for 45 min (35 °C, RH 75%). Finally, the fermented loaves were baked in a rotating oven (Sveba Dahlen, Fristad, Sweden) at 200 °C for 15 min. For steamed whole-grain wheat bread, the loaves were brought to a steaming pot and cooked for 15 min with the lid closed. For whole-grain wheat bread with no added yeast, the dough was immediately cut into three pieces (150 g each) after the first mixing without proofing, and the loaves were molded and baked in molds in the oven under the same conditions just described. The internal temperature of the bread was 95–99 °C for oven-baked bread and 88–92 °C for steamed bread. Three batches of bread were produced for each type of bread, and for each batch, three loaves of bread were prepared. Images of the bread types are provided in Figure 1. After cooling, bread from the same batch was cut into slices and ground in a coffee grinder (EGK 200, Rommelsbacher, Dinkelsbühl, Germany). The ground samples were sieved (3 mm), collected, and stored at −20 °C until further analysis. The moisture content of the bread samples was analyzed using the oven-dried method^25^ to report the data on a dry matter (DM) basis.

### Static In Vitro Digestion

The static in vitro digestion was carried out as previously described.^21^ Digestive fluids, including simulated salivary fluid (SSF, pH 7), simulated gastric fluid (SGF, pH 3), and simulated intestinal fluid (SIF, pH 7), were prepared according to the INFOGEST protocol.^26^ The activity of each enzyme and the bile acid concentration were determined before the assay^26^ and used to determine the quantity of enzymes or bile extract to be added during digestion.

The digestion included an oral phase, a gastric phase, and an intestinal phase, all performed at 37 °C. Briefly, the oral phase (2 min) started with the sample (5 g or 5 mL) being mixed with SSF containing α-amylase in a centrifuge tube. Next, in the gastric phase (2 h, pH 3), SGF containing pepsin was added. Finally, the intestinal phase (2 h, pH 7) started with adding SIF with bile extract, α-amylase, trypsin, and chymotrypsin to the tube. The total volume was 10 mL in the oral phase, 20 mL in the gastric phase, and 40 mL in the intestinal phase. At the end of the digestion, the supernatant of the digesta was collected for the following extraction and purification after centrifugation (18,500 rcf, 10 min). Duplicate digestion was carried out for each batch of bread, and a blank control was carried out to determine the folate level in the reagents.

### Extraction and Purification of Folate

A tri-enzyme method was applied to the extraction of folate as previously described.^27^ The extraction of a bread sample (1–2 g) included 10 min boiling in a water bath, 3 h of incubation with α-amylase and hog kidney conjugase, 1 h of incubation with protease, and 5 min boiling in a water bath to inactivate the enzymes. After cooling, the extract was centrifuged (18,500 rcf, 10 min), and the supernatant was collected and filtered through a 0.45 μm filter (4559 T, Pall Corporation, Port Washington, NY). The extraction of bread digesta (10 mL) started with 10 min boiling in a water bath, followed by 3 h of incubation with hog kidney conjugase, and 5 min boiling in a water bath. The extraction was carried out in duplicate for each batch of samples, and blank controls were used to distinguish the amount of endogenous folate versus that provided by the enzymes.

As previously described, the purification of folate extract was carried out using affinity chromatography^27^ with affinity agarose gel (Affi-Gel 10, Bio-Rad Laboratories, Richmond, CA) coupled with folate-binding protein (Scripps Laboratories, San Diego, CA). The purified folate extract was filtered through 0.2 μm filters (4927, Pall Corporation, Port Washington, NY), flushed with nitrogen, and stored at −20 °C for no more than 7 days before the folate quantification.

### Quantification of Folate

Quantification of folate was achieved using reversed-phase ultrahigh-performance liquid chromatography (UHPLC) coupled with fluorescence (FL) and photodiode array (PDA) detectors. The UHPLC method used in this study was modified from our previous research.^27^ The phosphate buffer (pH 2.2) as mobile phase A was replaced by Milli-Q water with 0.7% formic acid. Formic acid (0.7%) was added to mobile phase B (acetonitrile) to maintain the acidic environment during the analysis. The aim was to avoid the phosphate buffer mobile phase because the phosphate solution induces salt crystallization in the system, which can cause trouble with the maintenance of the equipment. Additionally, the phosphate-free mobile phase can be applied directly to a mass spectroscopy detector in the future.

A Kinetex 2.6 μm PS C18 LC column (150 × 2.1 mm; Phenomenex, Torrance, CA) was used to separate the folate vitamers. Gradient elution was applied as follows: 5% B, 0–0.5 min; 5–9.4% B, 0.5–3.8 min; 9.4–10.4% B, 3.8–5 min; 10.4–80% B, 5–5.3 min; 80% B, 5.3–5.8 min; 80–5% B, 5.8–5.9 min; and 5% B, 5.9–6.9 min. Samples were kept in a dark autosampler at 4 °C, with a flow rate of 0.6 mL/min. The column temperature was set at 30 °C.

Folates were quantified using a combination of FL and PDA detectors. 5-HCO-H_4_folate and PGA were detected by the PDA detector at a wavelength of 290 nm, while 5,10-CH^+^-H_4_folate was detected by the PDA detector at a wavelength of 360 nm. 10-HCO-H_2_folate and 10-HCO-PGA were detected by the FL detector at an excitation wavelength of 360 nm and an emission wavelength of 465 nm. H_4_folate and 5-CH_3_H_4_folate were detected by the FL detector at an excitation wavelength of 290 nm and an emission wavelength of 356 nm. The identification of folates was achieved by comparing the retention time of the samples’ peaks to the calibrants’ peaks. Additionally, the ultraviolet spectra of the calibrant and sample peaks were compared to confirm the vitamers. Quantification was performed using the external calibration curves.

### Validation of the UHPLC Method

Validation of the UHPLC method was performed by studying the linearity, sensitivity, precision, and recovery of the method. The concentrations of individual vitamers in the calibrant mixture ranged from 8 to 48 ng/mL (5,10-CH^+^-H_4_folate was prepared separately), except for 10-HCO-H_2_folate (16–96 ng/mL). Three calibration curves were constructed at three different dates to test the linearity. Based on our previous research,^27^ the linearity was tested at 80–1440 pg/injection (injection volume 10–30 μL) for each vitamer, except for 5-HCO-H_4_folate, 5,10-CH^+^-H_4_folate, and 10-HCO-H_2_folate (320–1440, 160–1440, and 160–2880 pg/injection, respectively), because of their high limit of limit of quantification (LOQ). Sensitivity was shown by the limit of detection (LOD) and LOQ, which were calculated by estimating the peak height that was 3 times and 10 times the background noise, respectively. Each concentration was injected in triplicate. The chromatographic parameters shown in Table S1 were obtained with Empower 2 software (Waters, Milford, MA).

The precision was estimated by the intra- and interday analysis of the calibrant solution. For intraday precision, a triplicate injection of 20 μL of the calibrant mixture was performed. For interday analysis, the injections were performed in three different weeks. The calibrant mixture was injected at a volume of 20 μL three times each day. The relative standard deviation (RSD, %) was calculated to show the method’s precision. The recovery test was performed by spiking three different matrices (5 mL of CHES/HEPES buffer, 1 g of whole-grain wheat flour, and 0.5 g of faba bean flour) with 140 ng of each vitamer (280 ng for 10-HCO-H_2_folate) in triplicate. The spiking levels were selected to be close to what is typically found in cereal products.^27^ The folate extraction, purification, and quantification were carried out as described in earlier sections.

### Stability Tests for 5-CH_3_H_4_folate during In Vitro Digestion

**Figure 2:**
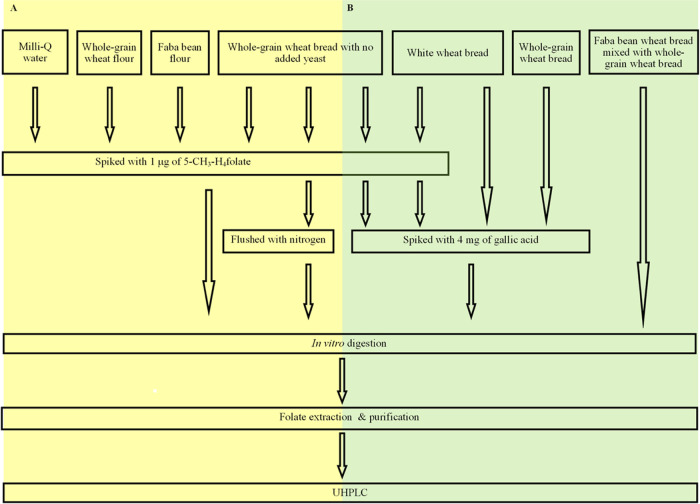
Procedures used in the spiking experiments to study the stability of 5-CH_3_-H_4_folate during in vitro digestion. (A) Samples were spiked with only 5-CH_3_-H_4_folate. (B) Samples were spiked with GA, except for faba bean wheat bread, where half of the faba bean wheat bread was replaced by whole-grain wheat bread.

To investigate the stability of 5-CH_3_-H_4_folate in different matrices as well as the possible protective effect of gallic acid (GA) on 5-CH_3_-H_4_folate during digestion, 5 g of water, whole-grain wheat flour, faba bean flour, and selected bread types were spiked with 1 μg of 5-CH_3_-H_4_folate, 4 mg of GA, or both at the beginning of the in vitro digestion as illustrated in Figure 2, except for faba bean wheat bread mixed with whole-grain wheat bread in a ratio of 1:1. The amount of added GA was based on the free phenolic content in 5 g of faba bean wheat bread. In addition, to study the effect of elimination of oxygen on the stability of 5-CH_3_-H_4_folate during digestion, whole-grain wheat bread with no added yeast and spiked with 1 μg of 5-CH_3_-H_4_folate was flushed with nitrogen for 10 s at the beginning of each of the oral, gastric, and intestinal phases. Whole-grain wheat bread was spiked with GA only because it contained a large amount of endogenous 5-CH_3_-H_4_folate. The digestion and folate analysis were carried out as previously described.

### Determination of Phenolic Content

Phenolic compounds were extracted in three separate fractions (soluble free, soluble esterified, and bound) according to Li et al.^28^ with modifications, and total phenolics in each fraction were determined spectrophotometrically using the Folin–Ciocalteu method. Three types of bread were selected based on the ingredients: faba bean wheat bread, whole-grain wheat bread with no added yeast, and white wheat bread. Approximately 0.1 g of sample was extracted three times in 1.5 mL of 80% ethanol solution with sonication for 10 min. Between extractions, the sample was centrifuged (4800 rcf) for 10 min. The supernatants were combined in a 5 mL volumetric flask which was filled to the mark. Residue was saved for the extraction of bound phenolics. For the determination of soluble free and esterified phenolics, we used 3.5 and 1.5 mL, respectively, of 80% ethanol extracts, which were evaporated to dryness under nitrogen at 35 °C, and the dried fraction was kept at −20 °C until use in analyses for free phenolics.

For soluble esterified phenolics, 0.4 mL of NaOH (2 M) was added to the dry fraction, followed by incubation with shaking on an orbital shaker at room temperature for 16 h. After incubation, 0.08 mL of HCl (12 M) and a small amount of solid NaCl (to avoid emulsion) were added. The free and esterified phenolics were extracted with ethyl acetate three times, and the combined ethyl acetate fraction was then evaporated to dryness under nitrogen at 35 °C. This dried fraction was stored at −20 °C until use in analyses as free and esterified phenolics.

Ethanol insoluble and ester-bound phenolics were extracted in the residue from the ethanol extraction. In brief, the solid was mixed with 4 mL of NaOH (2 M) and incubated for 16 h with shaking on an orbital shaker at room temperature. After centrifugation for 15 min (4800 rcf), the supernatant was mixed with 0.6 mL of HCl (12 M) and a small amount of solid NaCl. Ethyl acetate was applied to extract the bound phenolics, and the extraction was repeated twice. The extracts were combined and evaporated to dryness under nitrogen at 35 °C. This dried fraction was kept at −20 °C until use in analyses as ester-bound phenolics.

Before the spectrophotometric determination, the fractions were re-dissolved in 0.5 mL of 10% methanol solution and mixed with 1 mL of diluted (1:10) Folin–Ciocalteu reagent and 0.8 mL of 7.5% Na_2_CO_3_ solution. The mixture was incubated in the dark at room temperature for 30 min. The absorbance was measured at a wavelength of 765 nm, and the standard curve was constructed by GA solution with different concentrations. Triplicate analysis was carried out for each sample, and the phenolic content was determined as gallic acid equivalents (GAE).

### Statistical Analysis

The folate content was expressed as the mean ± standard deviation (μg/100 g, *n* = 3) on a DM basis. The phenolic content of the bread was expressed as the mean ± standard deviation (μg/100 g GAE, *n* = 3) on a DM basis. The mean values were obtained from six data points (triplicate baking process × duplicate folate analysis), except for the means (two data points, duplicate folate analysis) obtained from the stability test for 5-CH_3_-H_4_folate. Data visualization and analysis were performed using the R Studio platform. One-way analysis of variance and Tukey’s honestly significant difference post hoc test were applied for multigroup comparisons.

## Results and Discussion

### Performance of the UHPLC Method

The results of the method validation are shown in Table 2. The chosen concentration ranges (80–1440, 320–1440, 160–1440, and 160–2880 pg/injection) showed good linearity for all vitamers (*R*^2^ > 0.9). All seven vitamers were separated well in the chromatogram (Figure S1). Overall, the vitamers had a higher LOD or LOQ than with our previous method,^27^ and 5-HCO-H_4_folate had the highest LOD and LOQ because of its wide peak shape (Figure S1). 5-CH_3_-H_4_folate and H_4_folate had the lowest LOD and LOQ, which is consistent with the results of our previous study.^27^ Unlike with our previous method,^27^10-HCO-H_2_folate was quantitated using the FL detector in this study, and good sensitivity for 10-HCO-H_2_folate was observed (Table 2). 10-HCO-H_2_folate exhibited fluorescent behavior similar to 10-HCO-PGA.^29^ Because the FL detector is more sensitive than the PDA detector, identifying and quantifying 10-HCO-H_2_folate were easier than with our previous method.^27^

**Table 2:** Method Validation Results^a^

folate vitamer (detectors used for the quantification)	linearity and sensitivity			intraday variability (RSD %, *n* = 3)		interday variability (RSD %, *n* = 3 × 3)		recovery (%, mean ± standard deviation,*n* = 3)		
	*R*^2^	LOD (ng/mL)	LOQ (ng/mL)	retention time	peak area	retention time	peak area	extraction buffer	whole-grain wheat flour	faba bean flour
10-HCO-H_2_folate (FL 360/460 nm)	0.964	7.3	24.3	0.21	3.28	1.52	10.66	85 ± 12	67 ± 10	49 ± 5
10-HCO-PGA (FL 360/460 nm)	0.991	5.5	18.3	0.27	6.16	0.57	9.06	118 ± 3	88 ± 3	87 ± 18
5-HCO-H_4_folate (UV 290 nm)	0.982	13.1	43.6	0.34	1.29	0.84	5.94	122 ± 12	102 ± 4	92 ± 7
5,10-CH^+^-H_4_folate (UV 360 nm)	0.998	7.2	23.9	0.11	3.49	0.92	8.62	119 ± 2	160 ± 7	163 ± 23
5-CH_3_-H_4_folate (FL 290/356 nm)	0.993	0.4	1.4	0.51	0.96	0.91	6.31	103 ± 2	81 ± 2	92 ± 4
H_4_folate (FL 290/356 nm)	0.986	0.7	2.3	0.36	1.10	0.92	3.65	86 ± 8	98 ± 3	83 ± 6
PGA (UV 290 nm)	0.974	6.4	21.4	4.77	2.14	3.64	10.17	95 ± 11	92 ± 6	83 ± 6
total folate								108 ± 4	94 ± 4	86 ± 6

a A linear range of 80–1440 pg/injection (the injection volume was 10–30 μL) was chosen for each vitamer, except for 5-HCO-H_4_folate, 5,10-CH^+^-H_4_folate, and 10-HCO-H_2_folate (320–1440, 160–1440 and 160–2880 pg/injection, respectively). The injection volume used to determine LOD (the limit of detection) and LOQ (limit of quantification) was 20 μL. RSD, relative standard deviation. The recovery test was carried out by spiking different matrices with 140 ng of each vitamer (280 ng for 10-HCO-H_2_folate), and recovery (%) was calculated by the following equation: 100 × (the folate amount of the spiked matrices—the folate amount of the unspiked matrices)/the amount of folate used for spiking.

The results of intraday and interday precision showed satisfactory reproducibility (RSD < 11%) of the method (Table 2). Good precision (RSD < 7%) was observed for H_4_folate, 5-HCO-H_4_folate, and 5-CH_3_H_4_folate. Intraday precision was better than interday precision. Good recovery of total folate (> 85%) was observed, and nearly all the folate vitamers (except 10-HCO-H_2_folate) showed over 80% of recovery both with and without food matrices during the analysis (Table 2). However, a low recovery of 10-HCO-H_2_folate was observed in whole-grain wheat flour (67%) and faba bean flour (49%). Conversely, about 160% of 5,10-CH^+^-H_4_folate was recovered in these food matrices (Table 2). This is probably due to folate interconversion during the folate analysis. With the presence of antioxidants from food matrices, 10-HCO-H_2_folate can be reduced to 10-HCO-H_4_folate and then converted to 5,10-CH^+^-H_4_folate, which is more stable in an acidic environment.^11^

Overall, the validation data demonstrated that the current method was suitable for quantifying seven folate vitamers in cereal and legume matrices. Additionally, due to applying a column that was different from our previous method, the gradient elution time was shortened to 7 min, compared to 13 min with the previous system.^27^ However, the buffer system that we used previously seemed to stabilize folates better during the analysis than the current system, demonstrated by the better precision of the previous method (RSD < 5%) than the current one (RSD < 11%).

### Folate Content and Folate Bioaccessibility in Bread

Faba bean wheat bread had the highest total folate content, followed by steamed whole-grain wheat bread, whole-grain wheat bread, white wheat bread, and whole-grain wheat bread with no added yeast (Table 3). In bread digesta, the highest folate content was observed in faba bean wheat bread, while the lowest was found in white wheat bread, resulting in a folate bioaccessibility of 76 and 44%, respectively. Whole-grain wheat bread and steamed whole-grain wheat bread had similar folate bioaccessibility, around 60%, and no significant difference (*p* > 0.05) was found between the folate levels in their digesta. Whole-grain wheat bread with no added yeast had the highest folate bioaccessibility of 96%, although its folate level was low in both bread and bread digesta.

**Table 3:** Folate Contents and Folate Bioaccessibility of Bread^a^

bread	total folate content (μg/100 g DM)		folate bioaccessibility (%)
	before digestion	after digestion	
whole-grain wheat bread (WB)	53.3 ± 9.4cd	34.7 ± 6.1e	66 ± 12b
steamed whole-grain wheat bread (SB)	61.8 ± 7.2bc	37.2 ± 3.0e	61 ± 10bc
whole-grain wheat bread with no added yeast (WNB)	25.2 ± 4.2f	23.4 ± 1.0f	96 ± 21a
white wheat bread (WWB)	47.6 ± 1.7d	20.8 ± 2.2f	44 ± 4c
faba bean wheat bread (FWB)	89.7 ± 3.8a	68.1 ± 2.9b	76 ± 6ab

a Values are expressed as mean ± standard deviation. The standard deviations represent the variation among triplicate baking processes. Statistical analysis was carried out for total folate content and folate bioaccessibility separately, and values with different letters differ significantly (*p* < 0.05).

**Figure 3:**
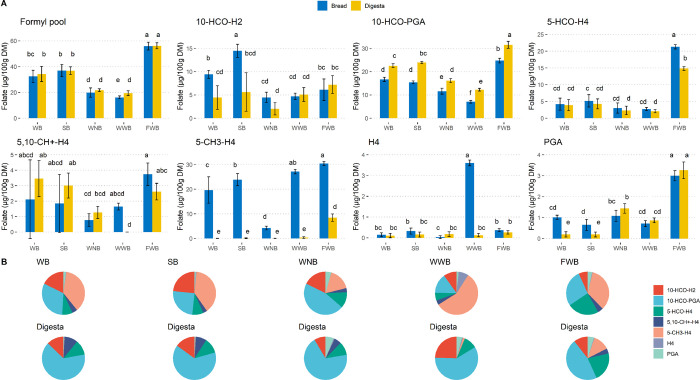
Contents (A) and distributions (B) of individual vitamers in bread and bread digesta. The error bars represent standard deviations among triplicate baking processes. Statistical analysis was carried out for each bar plot, and bars with different letters differed significantly (*p* < 0.05). WB, whole-grain wheat bread; SB, steamed whole-grain wheat bread; WNB, whole-grain wheat bread with no added yeast; WWB, white wheat bread; FWB, faba bean wheat bread.

The contents and distribution of individual vitamers in bread and bread digesta are summarized in Figure 3. In all of the bread types with added yeast, 5-CH_3_-H_4_folate was the main vitamer (34–57%). However, the loss of this vitamer was prominent during in vitro digestion. In the digesta of whole-grain wheat bread, steamed whole-grain wheat bread, and white wheat bread, the contribution of 5-CH_3_-H_4_folate to the total folate became negligible. 10-HCO-PGA, which was the predominant folate (46%) in whole-grain wheat bread with no added yeast and the second most dominant folate (15–31%) in all the other bread samples, became the main folate in all of the bread digesta (46–69%). The content of 5-HCO-H_4_folate in faba bean wheat bread was 21.3 ± 0.7 μg/100 g DM, which was 4–7.6 times the content of 5-HCO-H_4_folate in other bread samples. Although the level of 5-HCO-H_4_folate decreased in all the bread samples during digestion, it was still one of the main vitamers (22%) in faba bean wheat bread digesta. The formyl folate pool, which was expressed as the sum of 10-HCO-H_2_folate, 10-HCO-PGA, 5-HCO-H_4_folate, and 5,10-CH^+^-H_4_folate in this study, accounted for 34–79% of the total folate in bread and was the largest group of folate vitamers in bread digesta (82–99%). Although there is no formyl group in 5,10-CH^+^-H_4_folate, this vitamer is the interconvertible intermediate form of 5-HCO-H_4_folate and 10-HCO-H_4_folate in an acidic environment.^10^ Therefore, the sum of these vitamers reflects the level of formyl folates in food matrices. Overall, the changing patterns of individual vitamers during in vitro digestion are consistent with our previous studies.^21^ These patterns were: (1) the interconversion among formyl folates; (2) the decrease of reduced folates (5-HCO-H_4_folate, 5-CH_3_-H_4_folate, and H_4_folate); and (3) the high stability of the oxidized folates (mainly 10-HCO-PGA) and the formyl pool.

In bread matrices, the formyl pool as a whole was bioaccessible, but 5-CH_3_-H_4_folate was not. As shown in Figure 3, there was interconversion among formyl folates during digestion, but the sum of these vitamers remained stable in digesta. Conversely, the loss of 5-CH_3_-H_4_folate was significant during in vitro digestion. Similar phenomena were observed in other studies. O’Broin et al.^30^ used the growth of *Lactobacillus casei* to test the nutritional stability of different folate vitamers and reported that 5-HCO-H_4_folate and 10-HCO-H_4_folate were much more stable than 5-CH_3_-H_4_folate. In addition, Delchier et al.^31^ found that 5-HCO-H_4_folate and 10-HCO-H_4_folate in vegetables were stable at 45 and 60 °C with the presence of oxygen, while 5-CH_3_-H_4_folate was almost entirely degraded in 2 h. Therefore, an effective method for improving the bioaccessibility of folate would be to increase the proportion of formyl folates in food matrices.

### Effect of Ingredients on Folate Bioaccessibility in Bread

Previously, we reported that whole-grain wheat toast had better folate bioaccessibility and a higher content of bioaccessible folate (94%, 13 μg/100 g fresh matter) than white wheat toast (79%, 9 μg/100 g fresh matter).^21^ However, these types of bread were commercial, and we could not be sure that the ingredients caused the observed discrepancy. Here, the baking experiment confirmed that using whole-grain flour instead of refined flour can improve both folate content and folate bioaccessibility in bread. Whole-grain wheat bread had a higher total folate content than white wheat bread, and, in particular, whole-grain wheat bread had a larger formyl pool. Wheat bran has been found to be rich in formyl folates,^32^ and these vitamers were quite bioaccessible in our study. In addition, a considerably higher content (*p* < 0.05) of 5-CH_3_-H_4_folate was observed in white wheat bread than in whole-grain wheat bread. However, this vitamer was not stable, and its instability decreased the bioaccessibility of folate. Whole-grain wheat bread has been shown to have better antioxidant capacity than white bread.^33^ However, our results showed that the loss of 5-CH_3_-H_4_folate was complete in both whole-grain wheat bread and white wheat bread, indicating that the role of antioxidants in whole-grain flour can be negligible in protecting 5-CH_3_-H_4_folate in bread.

The inclusion of faba bean flour could partially protect 5-CH_3_-H_4_folate in the bread matrix from degradation during digestion. Legumes have become popular in recent years, and the inclusion of faba bean flour in baking is a promising way of improving the protein content in bread.^34^ Because faba bean flour is rich in folate,^35^ it could also be an effective way to enhance folate content and its bioaccessibility in bread. As shown in Table 3, the inclusion of faba bean flour increased the total folate content by 89% and the folate bioaccessibility by 73% compared to white wheat bread. Additionally, the bioaccessible folate content in faba bean wheat bread (68.1 ± 2.9 μg/100 g DM) was more than 3 times that in white wheat bread (20.8 ± 2.2 μg/100 g DM). Primarily, while there was no significant difference between the contents of 5-CH_3_-H_4_folate in white wheat bread and faba bean wheat bread, there was a considerably (*p* < 0.05) higher content of 5-CH_3_-H_4_folate observed in faba bean wheat bread digesta than white wheat bread digesta (Figure 3). Faba bean flour has been reported to be rich in polyphenols and exhibit excellent antioxidant capacity.^36^ Therefore, the antioxidants in faba bean flour could partially protect the 5-CH_3_-H_4_folate in faba bean wheat bread from degradation during baking or in vitro digestion, thus improving folate bioaccessibility.

### Effect of Baking Methods on Folate Bioaccessibility

Compared to oven baking, steaming had no significant effect on folate content and bioaccessibility in bread (Table 3). Steamed bread is commonly consumed in Asia and usually has a round shape.^37^ Steaming is a milder processing method than oven baking, and thus more folate was retained in steamed whole-grain wheat bread than oven-baked whole-grain wheat bread. Liang et al.^38^ reported that steaming caused more extensive folate loss than oven baking in wheat bread. However, they applied different protocols for preparing the bread, making the comparison less convincing. As for the folate bioaccessibility, there was no significant (*p* > 0.05) difference between the two types of bread, and the changes of individual folate vitamers were highly synchronized. Therefore, steamed whole-grain wheat bread and whole-grain wheat bread were rather similar from the perspective of folate content and bioaccessibility.

Baking with yeast enhanced the folate content in wheat bread but did not improve the bioaccessibility of folate. As shown in Table 3, the total folate content of whole-grain wheat bread with no added yeast was less than half that in whole-grain wheat bread with yeast fermentation. However, whole-grain wheat bread with no added yeast had significantly (*p* < 0.05) better bioaccessibility of folate (96%) than whole-grain wheat bread (66%), while the content of bioaccessible folate in the former bread (23.4 ± 1.0 μg/100 g DM) was significantly (*p* < 0.05) lower than the latter (34.7 ± 6.1 μg/100 g DM). The folate content derived from yeast, 28.1 and 11.3 μg/100 g DM in whole-grain wheat bread and its digesta, respectively, can be estimated by subtracting the total folate in whole-grain wheat bread with no added yeast from that in whole-grain wheat bread. The bioaccessibility of the yeast folate in whole-grain wheat bread was about 40%.

Contradictory results about the bioavailability of yeast folate have been found among researchers. Tamura & Stokstad^39^ reported around 60% folate availability of brewer’s yeast. Baker et al.^40^ claimed that yeast was a significant source of folate for young adults. However, Schertel et al.^41^ found that folate from concentrated yeast was only 8% available. The bioavailability of folate in foods is influenced by numerous factors, including the stability of folate, the polyglutamyl status of folate, the physiological condition of humans, and so on.^42^

Information about the vitamers (Figure 3) revealed that the relatively low folate bioaccessibility in whole-grain wheat bread was due to the low bioaccessibility of 5-CH_3_-H_4_folate during in vitro digestion. 5-CH_3_-H_4_folate is the main folate vitamer produced by many yeasts and bacteria^43,44^ that are widely present in baking. Whole-grain wheat bread had a significantly (*p* < 0.05) higher content of 5-CH_3_-H_4_folate than whole-grain wheat bread with no added yeast. However, this vitamer was not well bioaccessible in bread, which is worthy of attention.

### Possible Role of 5-Methyldihydrofolate (5-CH_3_-H_2_folate) in Folate Bioaccessibility

5-CH_3_-H_4_folate in bread matrices was unstable, and its stability affected the bioaccessibility of folate, especially when 5-CH_3_-H_4_folate was the main folate vitamer. However, the degradation pathway of this vitamer was not clear in this study. It is possible that 5-CH_3_-H_4_folate could have already been oxidized to 5-CH_3_-H_2_folate during baking.^45^ 5-CH_3_-H_2_folate can also be reduced to 5-CH_3_-H_4_folate with antioxidants.^46^ Additionally, 5-CH_3_-H_2_folate is much less stable than 5-CH_3_-H_4_folate under acidic environments,^47^ and thus, 5-CH_3_-H_2_folate could be quickly degraded during gastric digestion. If most of the 5-CH_3_-H_4_folate had been converted to 5-CH_3_-H_2_folate during baking, the low folate bioaccessibility in some types of bread could have been caused by the instability of 5-CH_3_-H_2_folate, as opposed to 5-CH_3_-H_4_folate, during digestion.

In contrast, the antioxidants in faba bean flour could have reduced 5-CH_3_-H_2_folate back to 5-CH_3_-H_4_folate during baking or digestion,^46^ resulting in the better folate bioaccessibility in faba bean wheat bread than in other wheat bread samples. Our previous study showed that only around 35% of 5-CH_3_-H_4_folate was degraded in the calibrant solution mixture during in vitro digestion.^21^ Therefore, 5-CH_3_-H_4_folate should not have been entirely degraded in the wheat bread. One plausible explanation is that there was already a certain amount of 5-CH_3_-H_2_folate in bread before digestion. Unfortunately, we could not quantitate 5-CH_3_-H_2_folate with our current method due to the use of antioxidants, which reduce 5-CH_3_-H_2_folate back to 5-CH_3_-H_4_folate during the analysis. Nevertheless, it can be concluded that the stability of 5-CH_3_-H_4_folate is crucial for the bioaccessibility of folate in foods, especially with foods that are rich in 5-CH_3_-H_4_folate.

### Stability of 5-CH_3_-H_4_folate Varied in Different Matrices during In Vitro Digestion

**Figure 4:**
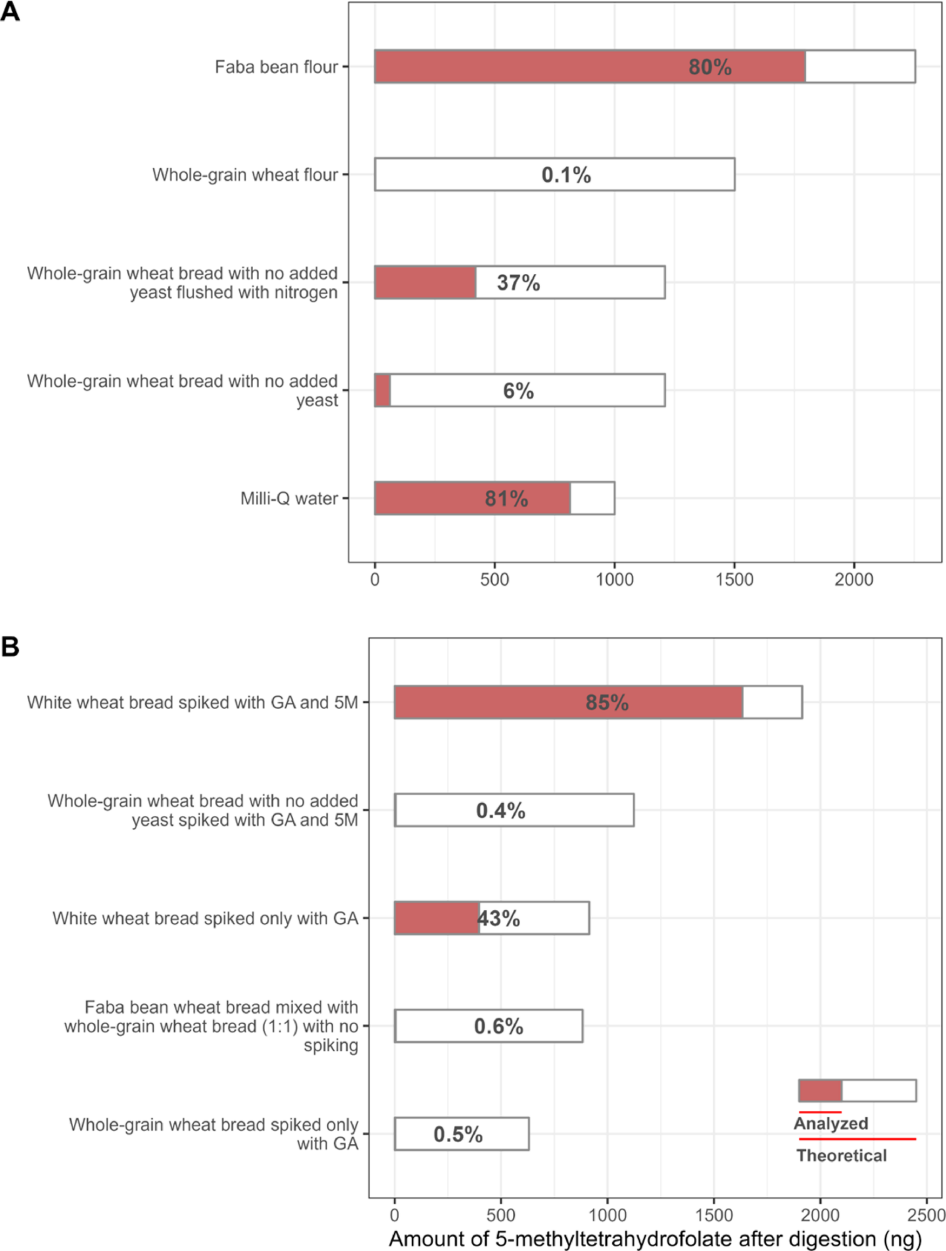
Amount (ng) of 5-CH_3_-H_4_folate in the digesta of different matrices. (A) 5 g of samples were spiked with 1 μg of 5-CH_3_-H_4_folate (5 M) only. (B) 5 g of samples were spiked with 4 mg of GA, except for faba bean wheat bread, where half of the faba bean wheat bread was replaced by whole-grain wheat bread. The recoveries (%) of 5-CH_3_-H_4_folate labeled in the bars were calculated by dividing the analyzed folate amount by the theoretical folate amount in the matrices; theoretical folate levels were calculated by summing up the amount of 5-CH_3_-H_4_folate in 5 g of the sample and the spiked amount of 5-CH_3_-H_4_folate. The content of the endogenous 5-CH_3_-H_4_folate in flour is shown in Table S2. Duplicate digestion was carried out for each sample. The results are expressed as means.

Because we could not be certain whether 5-CH_3_-H_4_folate or 5-CH_3_-H_2_folate was the main form of methyl folate in the bread before in vitro digestion, exogenous 5-CH_3_-H_4_folate was added to different matrices to study its stability. As shown in Figure 4A, 5-CH_3_-H_4_folate was unstable during in vitro digestion. Interestingly, the highest retention rate of 5-CH_3_-H_4_folate (81%) was observed in water, while the lowest was in whole-grain wheat flour (0.1%). Additionally, a low recovery of 5-CH_3_-H_4_folate (6%) was also observed in whole-grain wheat bread with no added yeast. However, the recovery was improved by 500% when the bread extract was flushed with nitrogen at the beginning of the different phases of in vitro digestion. It is likely that there are unknown factors in wheat matrices that could destroy 5-CH_3_-H_4_folate during in vitro digestion, and this phenomenon was related to oxidation. Ringling and Rychlik reported a 94% loss of 5-CH_3_-H_4_folate in wheat germ during in vitro digestion, even with added ascorbic acid, and speculated that the metal ions present in wheat germ could catalyze the oxidation of 5-CH_3_-H_4_folate.^14^ It has been shown that zinc and iron can accelerate the oxidation of 5-CH_3_-H_4_folate.^47^ Therefore, zinc and iron could also have catalyzed the degradation of 5-CH_3_-H_4_folate in our study.

Conversely, the retention of 5-CH_3_-H_4_folate in faba bean flour (80%) was comparable to that in water (81%). However, loss of 5-CH_3_-H_4_folate was greater for faba bean flour (460 ng) than water (186 ng). This indicates that unknown factors accelerating the degradation of 5-CH_3_-H_4_folate could also exist in faba bean matrices. Faba bean flour is rich in zinc and iron, but at the same time, it is also rich in phytate, which can chelate the metal ions and limit their activity.^48^ Therefore, the metal ions in faba bean matrices may not function well enough to catalyze the oxidation of 5-CH_3_-H_4_folate during in vitro digestion. A similar result was observed in our previous study, where the loss of 5-CH_3_-H_4_folate in faba bean matrices was less severe than that in cereal mactrices.^23^ Because oxidation played an essential role in the 5-CH_3_-H_4_folate loss during in vitro digestion, another hypothesis to explain the good stability of 5-CH_3_-H_4_folate in faba bean matrices is that these matrices had better antioxidant capacity than cereal matrices.

Ascorbic acid, which is secreted in human stomach with concentration ranging from 0.036 to 0.129 μmol/mL,^49^ has been shown to protect 5-CH_3_-H_4_folate during digestion. Chandra-Hioe et al. reported that the losses of 5-CH_3_-H_4_folate in bread matrices during in vitro digestion were minimized by adding ascorbic acid (0.05 μmol/mL) to the gastric digestion juice.^50^ However, they used a different in vitro digestion model where the digestion tubes were flushed with nitrogen. Conversely, an almost complete loss of 5-CH_3_-H_4_folate in wheat germ during in vitro digestion was reported, even with the addition of ascorbic acid (0.08 μmol/mL) in the gastric phase.^14^ In our previous study, 100 μmol/mL of ascorbic acid in the gastric phase was able to stabilize 5-CH_3_-H_4_folate in faba bean, oat, and rye flours during digestion, whereas 0.1 μmol/mL of ascorbic acid could not.^23^ The content of endogenous ascorbic acid in faba bean matrices is probably not high enough to stabilize 5-CH_3_-H_4_folate during digestion. In contrast, phenolic compounds are major contributors to the antioxidant capacity of many foods,^51^ and faba bean flour has been shown to possess good antioxidant activity that is highly correlated with its phenolic content.^52^

### Effect of Gallic Acid on 5-CH_3_-H_4_folate Stability during In Vitro Digestion

**Figure 5:**
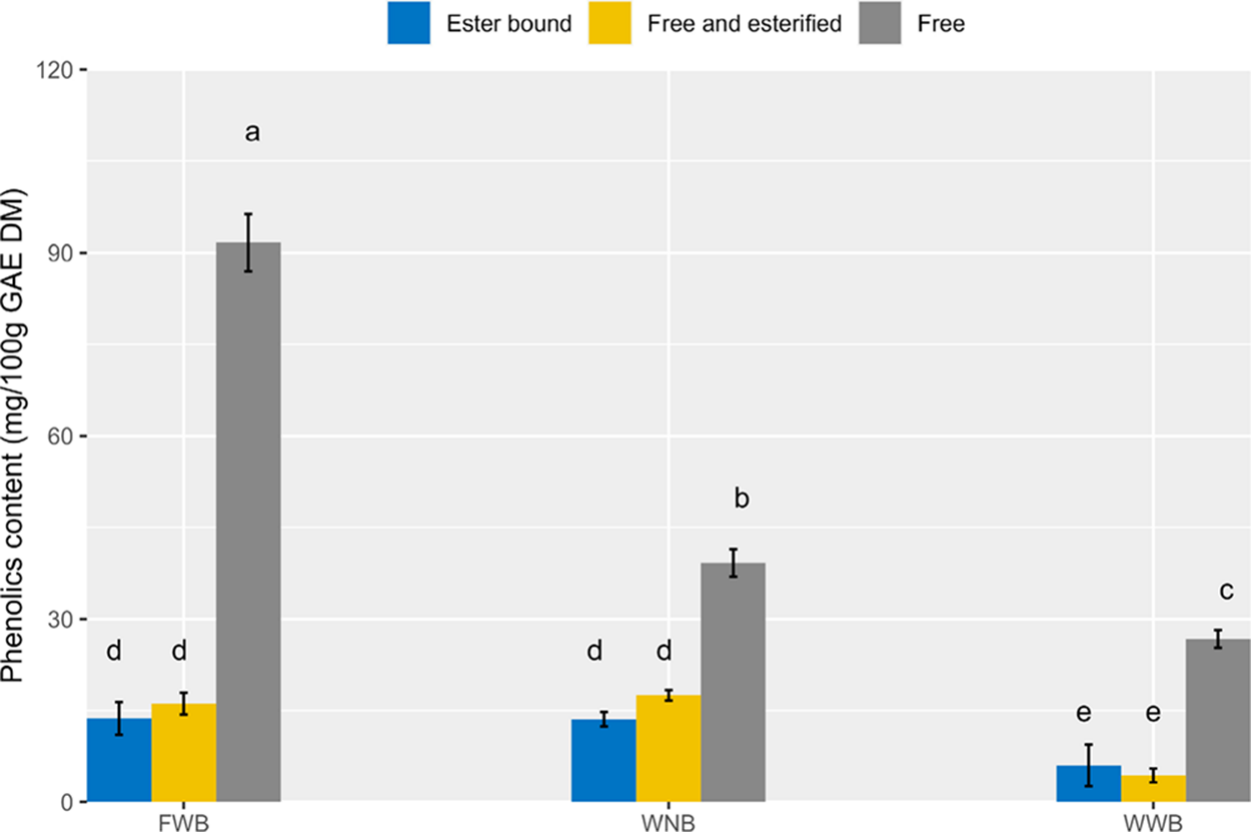
Phenolic contents (mg GAE/100 g DM) in FWB, WNB), and WWB. The error bars represent the standard deviation of triplicate baking processes. The results are expressed as mean ± standard deviation. Bars with different letters differed significantly (*p* < 0.05).

As shown in Figure 5, faba bean wheat bread had the highest free phenolic content, followed by whole-grain wheat bread with no added yeast and white wheat bread. However, the contents of free esterified and ester-bound phenolics were similar (*p* > 0.05) in faba bean wheat bread and whole-grain wheat bread with no added yeast, and white wheat bread had significantly (*p* < 0.05) lower contents of these two types of phenolics than the other bread. The results clearly show that the phenolic content, especially free phenolics, of faba bean wheat bread was significantly (*p* < 0.05) higher than that of the other bread.

A higher free phenolic level in faba bean wheat bread may protect 5-CH_3_-H_4_folate from oxidation during digestion. One study reported that the portion of free phenolics in wheat bread matrices correlates positively with the bioaccessibility of phenolics.^53^ Additionally, Lafarga et al. found that phenolics in faba beans, especially in cooked faba beans, were released during in vitro digestion, exhibiting great antioxidant activity.^54^ Therefore, a higher level of free phenolics in faba bean wheat bread could lead to higher bioaccessibility of phenolics and thus higher antioxidant capacity than in other bread samples. Although there were free phenolics present in wheat bread, it seems that these amounts were not enough to protect 5-CH_3_-H_4_folate from degradation during digestion.

To confirm the protective effects of phenolics during in vitro digestion, we spiked the wheat bread with GA and studied the stability of 5-CH_3_-H_4_folate during digestion. The amount of added GA was based on the free phenolic content in 5 g of faba bean wheat bread. The results (Figure 4B) showed that the added GA protected 5-CH_3_-H_4_folate in white wheat bread during in vitro digestion. When white wheat bread was spiked with GA, 43% of 5-CH_3_-H_4_folate was retained (396 ng), and thus the bioaccessibility of total folate was improved. Interestingly, the bioaccessibility of 5-CH_3_-H_4_folate in faba bean wheat bread was 28% (420 ng, estimation from Figure 3). The only difference between the preparation of white wheat bread and faba bean wheat bread was the ingredients, where half of the wheat flour was replaced by faba bean flour in faba bean wheat bread. This supports our hypothesis that phenolic compounds played a crucial role in retaining 5-CH_3_-H_4_folate in faba bean wheat bread during in vitro digestion, but probably only when they were in a sufficient amount and well bioaccessible.

To further confirm the protective effect of GA, white wheat bread was spiked with both GA and 5-CH_3_-H_4_folate, and in that case, the 5-CH_3_-H_4_folate recovery during in vitro digestion was 85%. Puthusseri et al.^55^ treated coriander plants with salicylic acid (a type of phenolic acid) solution and found that the folate level, as well as the folate bioaccessibility, in salicylic acid-treated coriander was improved compared to those in the normal group treated with tap water. Additionally, they found that salicylic acid reduced pro-oxidant status in the coriander. To the best of our knowledge, there has been no study investigating the relationship between phenolics and folates in vitro in the context of food matrices, and this topic warrants further research.

In contrast, the added GA did not protect 5-CH_3_-H_4_folate in whole-grain wheat matrices during in vitro digestion. As shown in Figure 4B, that was almost no recovery of 5-CH_3_-H_4_folate in whole-grain wheat matrices recorded despite spiking with GA. This again confirms that there are unknown factors in wheat matrices quickening the degradation of 5-CH_3_-H_4_folate during in vitro digestion, and these factors were mainly located in wheat bran. Given that our previous study reported the instability of 5-CH_3_-H_4_folate in rye and oat matrices during in vitro digestion,^23^ these unknown factors could also exist in rye and oat. Metal ions in cereal grains are mainly located in the aleurone layer of cereal bran,^56^ which further supports the hypothesis that the dramatic loss of 5-CH_3_-H_4_folate in wheat matrices during in vitro digestion could be caused by the catalyzing effect of metal ions. Even when half of the whole-grain wheat bread was replaced by faba bean wheat bread, after in vitro digestion, 5-CH_3_-H_4_folate was still unstable, and there was a recovery of merely 0.4%. It seemed that the pro-oxidant factors outweighed the protective factors in this case.

In summary, both ingredients and baking methods affected the bioaccessibility of folate in bread. The inclusion of whole-grain flour or faba bean flour during baking increased the folate content and the bioaccessibility of folate in the bread. Steamed whole-grain wheat bread and oven-baked whole-grain wheat bread were similar in terms of folate content and bioaccessibility. Baking with yeast significantly improved the folate level in bread but decreased folate bioaccessibility because of the instability of 5-CH_3_-H_4_folate. 5-CH_3_-H_4_folate was shown to play a critical role in the bioaccessibility of folate in bread, and further experiments confirmed that oxidation was one of the important reasons for the loss of 5-CH_3_-H_4_folate during in vitro digestion. Additionally, our study indicated that the pro-oxidant factors in wheat bran accelerated the degradation of 5-CH_3_-H_4_folate during in vitro digestion, while GA was able to protect 5-CH_3_-H_4_folate from degradation in white wheat bread. Therefore, the stability of 5-CH_3_-H_4_folate in food matrices during in vitro digestion resulted from a trade-off between the protective factors and pro-oxidant factors, which would eventually affect the bioaccessibility of folate. Other factors affecting the stability of 5-CH_3_-H_4_folate during digestion, such as pH, should also be investigated.

To improve folate bioaccessibility in bread (or other food products), we could: (1) raise the contribution of formyl folate pool to the total folate in foods or (2) include ingredients rich in antioxidants during processing. The minimum amount of antioxidant for stabilizing 5-CH_3_-H_4_folate during digestion should be clarified, and the degradation pathway of 5-CH_3_-H_4_folate during digestion should be elucidated in the future. Pro-oxidant factors accelerating the degradation of 5-CH_3_-H_4_folate should also be clarified in the future. Finally, in vivo studies should be carried out to test the findings from bioaccessibility studies.

## Supporting Information Available

Performance of the UHPLC-PDA/FL system (Table S1); folate content in the flours used for the baking (Table S2); and examples of UHPLC chromatograms in this study (Figure S1) (PDF)
