# Supplementary material for: 5-Methyltetrahydrofolate Is a Crucial Factor in Determining the Bioaccessibility of Folate in Bread
Source: J Agric Food Chem. 2022 Oct 7;70(41):13379–90. doi: 10.1021/acs.jafc.2c03861 (PMC9585583; doi:10.1021/acs.jafc.2c03861)
Supplement: Supplementary file 1 — jf2c03861_si_001.pdf [file jf2c03861_si_001.pdf]

Supporting Information

5-Methyltetrahydrofolate is a Crucial Factor in Determining the Bioaccessibility of Folate in Bread

Fengyuan Liu*, Minnamari Edelmann, Vieno Piironen, Susanna Kariluoto

Department of Food and Nutrition, University of Helsinki, Agnes Sjöbergin katu 2, FI-00014, Helsinki, Finland

*Corresponding author

E-mail address: phoyueen@outlook.com (F. Liu), minnamari.edelmann@helsinki.fi (M. Edelmann), vieno.piironen@helsinki.fi (V. Piironen), susanna.kariluoto@helsinki.fi (S. Kariluoto).

Table of Contents

Table S1. Performance of the UHPLC-PDA/FL system.

Table S2. Folate content (µg/100g in fresh weight) in the flours used for the baking.

Figure S1. UHPLC chromatograms from different detectors.

Table S1. Performance of the UHPLC-PDA/FL system

Vitamers	Retention factor	Number of theoretical plates	Selectivity	Resolution	Symmetry factor
**10-HCO-H_2folate**	10.4	10345	1.2	2	1
**10-HCO-PGA**	12	5287	1.2	2.2	0.9
**5-CH_3-H_4folate**	6.1	6044	1.2	3.5	1.1
**5-HCO-H_4folate**	13.1	2784	1.3	3.6	1.2
**5,10-CH^+-H_4folate**	9.6	16339	1.1	1.5	1.2
**H_4folate**	5	5908	1.8	7.4	1.1
**PGA**	14.3	31276	1.1	1.7	1.1

Table S2. Folate content (µg/100g in fresh weight) in the flours used for the baking. Results are expressed as mean ± standard deviation, n = 3 (triplicate analysis)

Folate	Whole-grain wheat flour	Wheat flour	Faba bean flour
**10-HCO-H_2folate**	0.1 ± 0.2	0.5 ± 0.4	16.1 ± 1.3
**10-HCO-PGA**	10.5 ± 0.3	7.2 ± 0.9	20.7 ± 1.0
**5-CH_3-H_4folate**	10.0 ± 0.2	3.4 ± 0.1	25.1 ± 0.5
**5-HCO-H_4folate**	6.9 ± 0.5	2.7 ± 0.4	37.0 ± 4.8
**5,10-CH^+-H_4folate**	8.0 ± 0.3	1.9 ± 0.1	18.3 ± 0.7
**H_4folate**	3.9 ± 0.4	2.0 ± 0.2	33.6 ± 0.1
**PGA**	3.8 ± 1.4	0.2 ± 0.3	9.7 ± 3.7
**Total**	43.1 ± 1.9	18.0 ± 0.4	160.5 ± 1.8

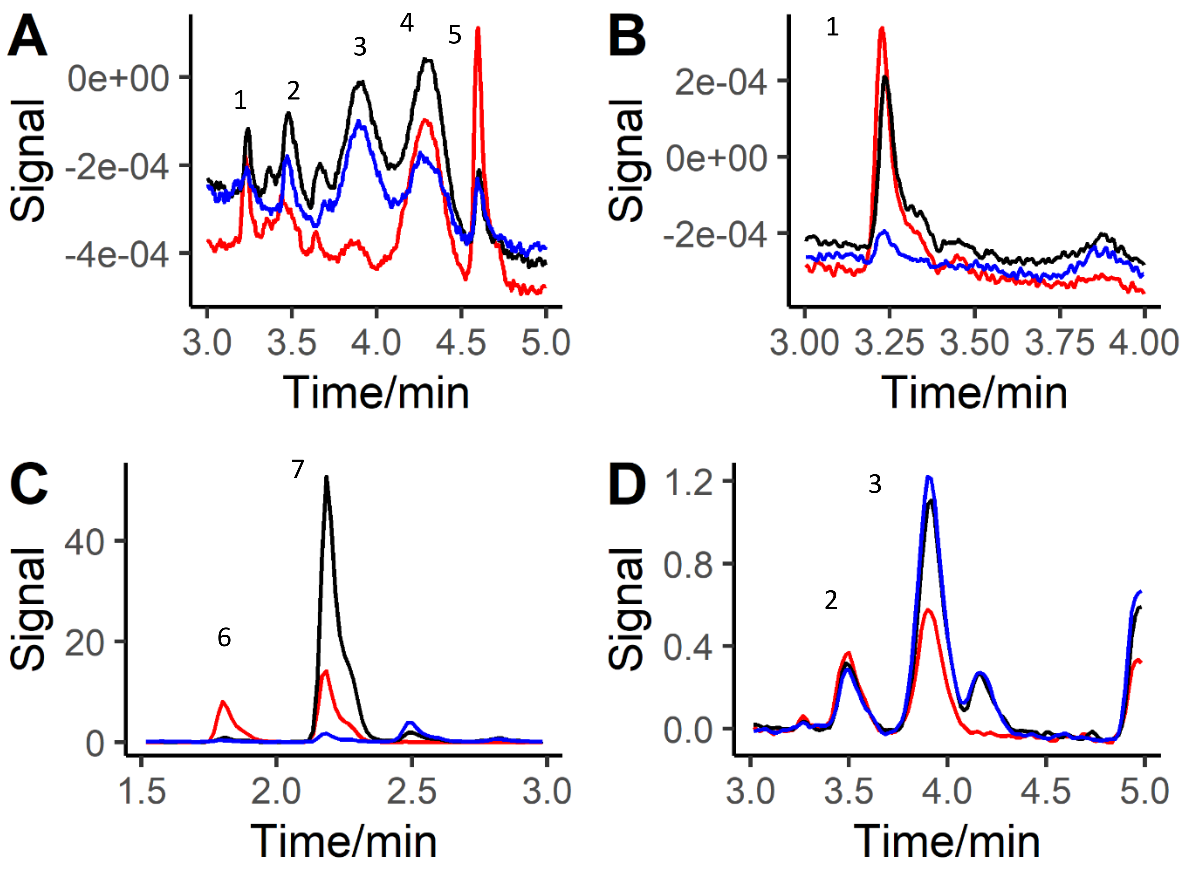

Figure S1. UHPLC chromatograms from different detectors (red: calibrants, black: steamed whole-grain wheat bread, blue: steamed whole-grain wheat bread digesta). A, PDA detector (290 nm); B, PDA detector (360 nm); C, FL detector (excitation wavelength: 290 nm; emission wavelength: 356 nm); D, FL detector (excitation wavelength: 360 nm; emission wavelength: 465 nm); vitamer peaks were labelled with numbers: 1, 5,10-CH^+-H_4folate; 2, 10-HCO-H_2folate; 3, 10-HCO-PGA; 4, 5-HCO-H_4folate; 5, PGA; 6, H_4folate; 7, 5-CH_3-H_4folate.
